# Association of the Planetary Health Diet with dementia risk and brain pathology

**DOI:** 10.1002/alz.71327

**Published:** 2026-04-01

**Authors:** Rongrong Yang, Jiao Wang, Abigail Dove, Sakura Sakakibara, Puja Agarwal, David A. Bennett, Weili Xu

**Affiliations:** ^1^ Public Health Science and Engineering College Tianjin University of Traditional Chinese Medicine Tianjin China; ^2^ Department of Neurobiology Aging Research Center Care Sciences and Society Karolinska Institutet Stockholm Sweden; ^3^ National Clinical Research Center for Geriatrics West China Hospital Sichuan University Chengdu China; ^4^ Rush Alzheimer's Disease Center Rush University Medical Center Chicago Illinois USA

**Keywords:** brain pathology, cognitive function proximate to death, dementia, longitudinal cohort study, Planetary Health Diet

## Abstract

**BACKGROUND:**

The Planetary Health Diet (PHD), proposed by the EAT‐Lancet Commission, promotes human and environmental health, yet its relevance to dementia and brain pathology remains unclear.

**METHODS:**

In the Rush Memory and Aging Project, 926 dementia‐free adults (mean age = 79.4) were followed for up to 20 years. Baseline diet was assessed using a >142‐item food‐frequency questionnaire, and PHD adherence was categorized into tertiles. Dementia and Alzheimer’s dementia (AD) were clinically diagnosed. Among 581 decedents, brain autopsies quantified Alzheimer’s disease, vascular, and other pathologies. Cognitive function proximate to death was measured via a standardized neuropsychological battery.

**RESULTS:**

Over a median 7.5‐year follow‐up, 317 participants developed dementia. High PHD adherence was associated with lower risks of all‐cause dementia (hazard ratio [HR] = 0.71) and AD (HR = 0.70), reduced Alzheimer’s disease pathologies, a 2.17‐year delay in dementia onset, and better end‐of‐life cognition.

**CONCLUSION:**

High PHD adherence may support healthier brain aging and cognitive resilience.

## Highlights

- Higher Planetary Health Diet (PHD) adherence lowers dementia risk.
- PHD adherence is associated with reduced Alzheimer's disease pathologies.
- PHD adherence relates to better cognitive function near end of life.
- Results suggest that sustainable diets may influence long‐term brain health.
- Findings highlight diet as a modifiable factor for dementia prevention.

## 1 BACKGROUND

Dementia affects more than 55 million individuals worldwide, posing major public health and societal challenges.^1^ In the absence of curative treatments, modifiable lifestyle factors, particularly diet, have gained attention as potential strategies for preventing Alzheimer's disease and delaying neurodegeneration.^2^, ^3^ Several dietary frameworks with an emphasis on optimizing individual health, such as the Mediterranean diet, DASH (Dietary Approaches to Stop Hypertension) diet, and the Mediterranean‐DASH Intervention for Neurodegenerative Delay (MIND) diet, have been linked to better cognitive outcomes and reduced risk of dementia.^4^, ^5^, ^6^

In 2019, the EAT‐Lancet Commission introduced the Planetary Health Diet (PHD) as a global reference diet that aims to simultaneously promote human health and safeguard planetary boundaries.^7^ This dietary model emphasizes a predominantly plant‐based dietary pattern that includes whole grains, fruits, vegetables, legumes, nuts, and unsaturated oils, while limiting red meat, added sugars, and highly processed foods.^8^ These dietary shifts are intended to reduce greenhouse gas emissions, land use, and freshwater consumption associated with food production. By shifting away from resource‐intensive animal‐based products and promoting diverse, plant‐forward eating, the PHD aims to support both planetary boundaries and long‐term population health. A growing literature has highlighted the association between the EAT‐Lancet PHD and increased longevity and lower risk of cardiometabolic outcomes.^8^, ^9^, ^10^, ^11^ Although several studies have examined the relation of PHD to cognitive health with mixed results, to our knowledge, only one study so far has addressed this diet in relation to dementia within a Swedish population‐based cohort.^12^ Here we extend the results to another cohort study in the United States and further examine the relation of the PHD to common brain pathologies.

Moreover, the biological pathways linking diet and cognitive health remain poorly understood.^13^ Neuropathological hallmarks of dementia, including amyloid beta (Aβ), neurofibrillary tangles, Lewy bodies, hippocampal sclerosis, and both macro‐ and microvascular brain lesions, begin to accumulate years or even decades before the emergence of clinical symptoms.^14^ However, whether adherence to the PHD is associated with differences in brain pathology has not been examined to date.

Additional insights into biological mechanisms can come from integrating neuropathological data with cognitive data.^15^ Several recent studies have examined the association between PHD adherence and cognitive outcomes, with some reporting favorable effects on cortical thickness and slower decline in global cognition, memory, executive function, and attention.^16^, ^17^, ^18^, ^19^ However, findings remain inconsistent, and it is unclear whether any cognitive benefits are independent of or explained by underlying neuropathological burden.

Using data from the Rush Memory and Aging Project (MAP), a longitudinal community‐based cohort with up to 20 years of follow‐up, we aimed to: (1) assess the association between PHD adherence and dementia risk; (2) examine the relationship between PHD and postmortem neuropathological burden; and (3) investigate the association between PHD adherence and proximate‐to‐death cognitive function, and examine the role of neuropathological burden in this association.

## 2 METHODS

### 2.1 Study design and participants

Rush MAP is an ongoing longitudinal clinical–pathologic study of aging and dementia comprising older adults living in retirement communities, senior housing, and individual residences in the greater Chicago area.^20^ From 1997 onward, participants underwent annual clinical evaluations, cognitive testing, risk factor assessments, and blood collection. In 2004, a nutrition sub‐study was introduced, during which participants began completing a validated, self‐administered >142‐item food‐frequency questionnaire (FFQ) to assess dietary intake, with data collected every year thereafter.^13^

As of July 1, 2024, the MAP had enrolled a total of 2357 participants, of whom 1060 had complete FFQ dietary data that were quality checked and shared for this study. We excluded participants with prevalent dementia at the time of the first FFQ (*n* = 53) and those without follow‐up data primarily due to death and not yet in the study a full year (*n* = 81), yielding a final analytic sample of 926 dementia‐free individuals. During the follow‐up period, 705 participants died and 581 (82.41%) of these underwent brain autopsy and had approved data for neuropathological assessment at the time of this study (Figure S1).

### 2.2 Ethics

The protocol was approved by the institutional review board of Rush University Medical Center and conducted in accordance with the Declaration of Helsinki. All participants provided written informed consent and organ donation documentation under the Uniform Anatomical Gift Act.

### 2.3 Dietary assessment

Dietary intake was assessed using a self‐administered >142‐item FFQ, adapted from the validated Harvard FFQ for older Chicago residents.^21^ Participants were asked to report their usual frequency of consumption for standard portion sizes of various food items over the past year.^6^ Daily intakes of energy, nutrients, and food groups were estimated by multiplying the reported frequency of consumption for each food and beverage item by its standard portion size and corresponding nutrient composition. Multivitamin use was assessed as part of the FFQ, based on participants’ self‐reported frequency of supplement intake. The reliability and validity of the FFQ in the Chicago Health and Aging Project were previously established and published.^21^, ^22^

### 2.4 Assessment of the PHD and other dietary patterns

Adherence to the PHD was evaluated using the EAT‐Lancet diet score, constructed in accordance with the dietary targets outlined by the EAT‐Lancet Commission in 2019.^7^, ^8^, ^23^ The score was calculated based on each participant's first available dietary assessment. Reported food intakes were first converted into grams per day based on standard U.S. Department of Agriculture serving sizes, and food items were then grouped into specific dietary components (Table S1). The dietary components were classified as either “emphasized foods,” including vegetables, fruits, legumes, whole grains, nuts, fish, and unsaturated fats, or “limited foods,” such as red meat (beef and lamb), pork, poultry, eggs, dairy products, potatoes, and added sugars.^7^ The index comprised 14 food components, each assigned a score from 0 (nonadherence) to 3 (full adherence) according to intake relative to EAT‐Lancet recommendations (Table S2). Possible values for the EAT‐Lancet score therefore range from 0 to 42, with higher scores indicating greater overall adherence to the PHD.^23^ In our sample, EAT‐Lancet diet scores ranged from 6.0 to 32.0, in line with the score range in previous studies.^17^ For analysis, the scores were treated as both a continuous variable and categorized into tertiles of low (*n* = 358, score 6.0–16.0), moderate (*n* = 308, score 17.0–19.0), and high (*n* = 256, score 20.0–32.0) adherence. Because of tied values, the tertile group sizes were not strictly equal.

For comparison, we also included three established dietary pattern scores. The MIND diet score includes 15 components (10 brain‐healthy and 5 unhealthy) with a total score ranging from 0 to 15.^24^ The Mediterranean diet score includes 11 components scored 0–5 for a total of 0–55.^6^ The DASH diet score is based on 10 food and nutrient components, each scored 0–1 for a total of 0–10.^6^ These dietary scores have been described and validated in previous studies.^5^, ^13^

**RESEARCH IN CONTEXT**

1. **Systematic review**: We searched PubMed and Web of Science for studies evaluating the Planetary Health Diet (PHD) in relation to dementia, brain pathology, or late‐life cognitive outcomes. Prior literature on sustainable diets and neurodegeneration is extremely limited; no studies have examined PHD adherence in relation to dementia incidence or neuropathology, and evidence on diet and end‐of‐life cognitive function remains scarce.
2. **Interpretation**: Our findings indicate that a higher adherence to the PHD is associated with lower dementia risk and reduced Alzheimer's disease pathologies. These associations extend to cognitive function near the end of life, suggesting that a sustainable dietary pattern may influence both long‐term neurodegenerative processes and terminal cognitive decline.
3. **Future directions**: Future research should test causal mechanisms linking sustainable diets with neuropathology, evaluate PHD interventions in diverse populations, and examine biological pathways—such as inflammation, metabolic dysfunction, and vascular health—that may mediate the observed associations.

### 2.5 Assessment of all‐cause dementia and Alzheimer's dementia (AD)

Dementia and AD were assessed using a uniform, structured process including computerized scoring of cognitive tests, clinical judgment by a neuropsychologist, and diagnostic classification by a clinician.^25^ Dementia and AD were diagnosed in accordance with the criteria from the joint working group of the National Institute of Neurological and Communicative Disorders and Stroke and the AD and Related Disorders Association, taking into consideration normative cognitive function based on age, sex, and education.^26^

### 2.6 Assessment of cognitive function

Cognitive function was assessed using a comprehensive battery of 19 cognitive tests assessing multiple cognitive domains.^27^ Details of the cognitive assessment procedure have been described in a previous study.^25^ A global cognitive function score was calculated as the average of the *z*‐scores across all 19 tests.^25^ Higher *z*‐scores indicated better cognitive performance, whereas lower scores reflected poorer performance relative to the cohort mean at baseline. In addition to the global score, domain‐specific composite scores were derived for episodic memory, semantic memory, working memory, visuospatial ability, and perceptual speed (Supplementary Method 1). We used the last available scores prior to death for participants with brain autopsy.^28^

### 2.7 Assessment of brain pathologies

Brain removal, tissue sectioning, preservation, and standardized gross and microscopic examinations were conducted according to a standardized protocol.^29^Multiple brain regions for autopsied brain samples were evaluated for Alzheimer’s disease pathology by quantifying three key indices: global Alzheimer’s disease pathology, Aβ load, and neurofibrillary tangle density, each analyzed as a continuous variable.^27^, ^30^ Aβ load was quantified as a composite measure based on immunohistochemical analysis across multiple cortical regions.^31^ Neurofibrillary tangle burden was assessed using phosphorylated tau staining and summarized as the mean tangle density across selected brain regions.^32^ Global Alzheimer’s disease pathology was derived from Bielschowsky silver‐stained sections, based on aggregated counts of neuritic plaques, diffuse plaques, and tangles across five cortical areas. Additional neuropathological markers, including Lewy bodies,^29^ hippocampal sclerosis, gross and microinfarcts, and cerebrovascular disease (e.g., atherosclerosis, arteriolosclerosis, and cerebral amyloid angiopathy), were assessed and classified as present or absent.^33^, ^34^ Detailed assessment protocols are provided in Supplementary Method 2.

### 2.8 Assessment of other covariates

Information on participants’ demographic characteristics and lifestyle factors was collected at baseline, including age, sex, education, total energy intake, apolipoprotein E (*APOE*) ε4 status, Mini‐Mental State Examination (MMSE) score, alcohol consumption, physical activity, body mass index (BMI), vascular disease burden (claudication, stroke, heart conditions, and congestive heart failure), vascular risk factors (hypertension, diabetes, and smoking), and multivitamin use (Supplementary Method 3). Additional details about the data collection are available at the Rush AD Center Resource Sharing Hub (https://www.radc.rush.edu/).

### 2.9 Statistical analysis

Baseline characteristics of the study sample were compared across the low, moderate, and high PHD adherence groups. Categorical variables were analyzed using chi‐square (*χ* ^2^) tests, whereas continuous variables were assessed using one‐way analysis of variance (ANOVA) for normally distributed data or Kruskal–Wallis tests for skewed distributions.

Cox proportional hazards regression models were used to estimate hazard ratios (HRs) and 95% confidence intervals (CIs) for the association between adherence to the PHD and risk of incident dementia. Follow‐up time (years) was defined as the interval from baseline dietary assessment to the first occurrence of dementia diagnosis, death, or last available evaluation. Stratified analyses were conducted to assess potential effect modification by age (< 80 vs ≥80 years; cutoff based on the population median), sex (male vs female), and *APOE ε*4 genotype status (carriers vs non‐carriers). Laplace regression models were additionally applied to estimate median time (in years, with 95% CIs) to dementia onset across levels of dietary adherence. To examine associations between the PHD and postmortem brain pathologies, we used linear regression models for continuous outcomes and logistic regression models for dichotomous neuropathological markers. All models were first basic adjusted for age, sex, education, and total energy intake, followed by additional adjustment for *APOE ε*4, MMSE score, alcohol consumption, physical activity, BMI, vascular disease burden (including claudication, stroke, heart conditions, and congestive heart failure), vascular risk factors (including hypertension, diabetes, and smoking), and multivitamin use. Parallel analyses were conducted for the MIND, Mediterranean, and DASH diets to compare their associations with dementia risk.

To assess whether the association between PHD adherence and late‐life cognitive function was independent of brain pathologies, we examined the relationship between diet adherence and cognitive function proximate to death using regression models with and without adjustment for common brain pathologies. Changes in the regression coefficient (*β* estimate) were evaluated to determine the extent to which these neuropathological measures accounted for the observed association.

To examine the robustness of our findings, we conducted multiple sensitivity analyses. First, we applied a competing risks model (the Fine–Gray model) with death as the competing event. Second, to reduce the potential for reverse causality, we excluded participants who developed dementia within the first 3 years of follow‐up and repeated the main analyses. Third, missing covariate data were minimal (17 [1.84%] for BMI, 5 [0.54%] for *APOE ε*4, and 7 [0.76%] for alcohol consumption) and were addressed using multiple imputation by chained equations with five imputed datasets. Any *p*‐values less than 0.05 were considered statistically significant. All statistical analyses were performed using Stata SE 16.0.

### 2.10 Role of funders

The funders had no role in study design, data collection, analysis, interpretation, manuscript preparation, or publication decision.

## 3 RESULTS

### 3.1 Baseline characteristics

At baseline, the mean (SD) age of the 926 participants was 79.4 (7.1) years; 695 (75.1%) were female, and the mean education level was 14.94 (± 2.95) years. Participants with high adherence to the PHD were more likely to have higher levels of education, greater physical activity, and lower vascular disease burden, and were also more likely to use multivitamin supplements (Table 1). These individuals also reported lower overall energy intake but higher consumption of whole grains, vegetables, fruits, dairy products, fish, legumes, nuts, and unsaturated oils. In contrast, they consumed less pork, added sugar, and poultry.

**TABLE 1:** Characteristics of the study population by adherence to the PHD.

Characteristics	Low (*n* = 355)	Moderate (*n* = 306)	High (*n* = 255)	*p‐*value
Follow‐up time, y	6.11 (3.04, 11.85)	7.03 (3.38, 12.06)	8.23 (4.67, 11.50)	—
Age, y	79.11 ± 7.79	79.83 ± 6.74	79.40 ± 6.38	0.419
Female	281 (79.15)	218 (71.24)	196 (76.86)	0.037
Education, y	14.17 ± 3.09	15.24 ± 2.66	15.65 ± 2.85	<0.001
BMI, kg/m^2^	27.19 ± 4.67	26.75 ± 4.85	27.63 ± 4.97	0.101
*APOE ɛ*4 carriers	73 (20.45)	67 (22.04)	61 (23.83)	0.607
Physical activity, h/week	2.31 (0.58, 4.25)	2.50 (0.92, 4.65)	3.50 (1.67, 5.33)	<0.001
Vascular disease burden^a^	0.35 ± 0.65	0.31 ± 0.60	00.21 ± 0.48	0.014
Vascular risk factors^b^	1.07 ± 0.83	1.07 ± 0.80	1.00 ± 0.76	0.529
Total energy intake, kcal/day	1969.79 ± 465.45	1751.26 ± 466.07	1561.02 ± 555.25	<0.001
Alcohol consumption, serving/day	1.00 (1.00, 2.00)	2.00 (1.00, 3.00)	1.00 (1.00, 3.00)	<0.001
Multivitamin use	237 (66.39)	202 (65.80)	199 (77.73)	0.003
PHD components, g/day	13.67 ± 2.18	17.93 ± 1.78	22.02 ± 1.94	
Whole grains	23.69 ± 22.13	31.22 ± 25.80	40.27 ± 30.04	<0.001
Potatoes	58.90 ± 39.49	61.84 ± 40.27	60.02 ± 38.43	0.641
Vegetables	132.28 ± 77.34	185.23 ± 88.91	265.32 ± 100.08	<0.001
Fruits	94.01 ± 59.77	131.99 ± 60.73	169.73 ± 68.38	<0.001
Dairy	239.30 ± 252.54	258.81 ± 199.03	304.06 ± 209.14	0.001
Beef and lamb	31.65 ± 25.78	30.45 ± 22.50	31.94 ± 25.70	0.747
Pork	14.01 ± 16.18	12.31 ± 12.35	10.64 ± 9.99	0.007
Poultry	36.52 ± 21.45	28.48 ± 19.03	22.80 ± 19.19	<0.001
Eggs	10.48 ± 11.26	13.77 ± 10.57	13.25 ± 8.92	<0.001
Fish	13.96 ± 13.15	20.67 ± 15.12	31.02 ± 17.26	<0.001
Nuts	2.98 ± 4.35	4.30 ± 5.32	6.42 ± 6.87	<0.001
Legumes	4.09 ± 7.57	7.48 ± 11.15	14.13 ± 13.89	<0.001
Unsaturated oils	30.97 ± 13.79	33.26 ± 11.99	35.82 ± 11.25	<0.001
Added sugar	37.84 ± 27.79	32.93 ± 21.49	32.08 ± 19.57	<0.001
Death during follow‐up	274 (76.54)	241 (78.25)	190 (74.22)	0.532
Autopsy	222 (62.01)	198 (64.29)	161 (62.89)	0.831

*Note*: Values are mean ± standard deviation, *n* (%), or median (interquartile range).

Abbreviations: *APOE*, apolipoprotein E; BMI, body mass index; PHD, Planetary Health Diet.

The number of subjects with missing values was 17 (1.84%) for BMI, 5 (0.54%) for *APOE* ε4, and 7 (0.76%) for alcohol consumption.

^a^ Vascular disease burden was calculated using self‐reported data on four conditions: intermittent claudication, stroke, heart disease, and congestive heart failure.

^b^ Vascular risk factors were defined as the presence of hypertension, diabetes mellitus, and a history of smoking.

### 3.2 PHD and dementia risk

During the follow‐up period (median: 7.48 years, interquartile range [IQR]: 3.14–11.37 years), 311 participants (33.6%) developed all‐cause dementia and 301 (32.5%) were diagnosed with AD. In multiple‐adjusted Cox regression models, higher adherence to the PHD (as a continuous variable) was associated with a significantly reduced risk of dementia (HR [95% CI]: 0.97 [0.94–0.99]) and AD (HR: 0.96 [0.94–0.99]) (Table 2). Participants in the high adherence group had a 29% lower risk of all‐cause dementia (HR: 0.71 [0.52–0.95]) and a 30% lower risk of AD (HR: 0.70 [0.52–0.94]) compared with those in the low adherence group.

**TABLE 2:** HRs and 95% CIs for the association between adherence to the PHD and risk of all‐cause dementia and AD: Results from Cox regression models.

	Dementia			AD		
PHD	No. of cases	HR (95% CI)^a^	HR (95% CI)^b^	No. of cases	HR (95% CI)^a^	HR (95% CI)^b^
Continuous	311	0.97 (0.94–0.99)	0.97 (0.94–0.99)	301	0.96 (0.94–0.99)	0.96 (0.94–0.99)
Categorical (tertiles)						
Low	125	Ref.	Ref.	124	Ref.	Ref.
Moderate	104	0.79 (0.60–1.03)	0.78 (0.59–1.01)	98	0.76 (0.58–1.00)	0.74 (0.57–0.98)
High	82	0.70 (0.52–0.93)	0.71 (0.52–0.95)	79	0.69 (0.51–0.93)	0.70 (0.52–0.94)

Abbreviations: AD, Alzheimer’s dementia; CI, confidence interval; HR, hazard ratio; PHD, Planetary Health Diet.

^a^ Adjusted for age, sex, education, and total energy intake.

^b^ Model adjusted for age, sex, education, *APOE ε*4, Mini‐Mental State Examination score, alcohol consumption, physical activity, body mass index, vascular disease burden (including claudication, stroke, heart conditions, and congestive heart failure), vascular risk factors (including hypertension, diabetes, and smoking), multivitamin use, and total energy intake.

**FIGURE 1:**
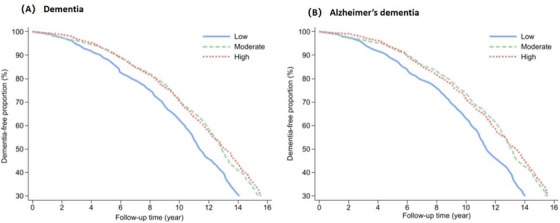
Dementia and Alzheimer’s dementia‐free proportion by PHD. Model adjusted for age, sex, education, *apolipoprotein E ε*4, Mini Mental State Examination score, alcohol consumption, physical activity, BMI, vascular disease burden (including claudication, stroke, heart conditions, and congestive heart failure), vascular risk factors including (hypertension, diabetes, and smoking), multivitamin use, and total energy intake.

In multivariable‐adjusted Laplace regression models, the onset of dementia was delayed by 2.17 years (95% CI: 0.54–3.79) among participants in the high compared to low adherence group. Likewise, the onset of AD was delayed by 2.51 years (95% CI: 1.09–3.93) in the high group and 1.89 years (95% CI: 0.76–3.02) in the moderate group, relative to the low adherence group (Figure 1, Table S3).

The PHD–dementia association remained significant after stratification by age, sex, and *APOE ε*4 genotype status. Furthermore, we found no significant interactions between these factors and PHD on the risk of dementia (Table S4–S6). Similar trends were observed for other dietary patterns (Table S7). Higher adherence to the MIND and Mediterranean diets was associated with a lower risk of all‐cause dementia, with multivariable‐adjusted HRs of 0.73 (95% CI, 0.54–0.99) and 0.67 (95% CI, 0.49–0.92), respectively, for the high versus low tertile. In contrast, adherence to the DASH diet showed no significant association with dementia risk (*HR* = 0.81; 95% CI, 0.61–1.07).

### 3.3 PHD and brain pathologies

Among the 581 participants who underwent brain autopsies, 232 (39.9%) had dementia, including 225 (38.7%) with AD. In multivariable linear regression models, higher adherence to the PHD was associated with significantly lower Alzheimer’s disease‐related brain pathology. Compared with participants in the low PHD tertile, those in the high tertile had significantly lower levels of global Alzheimer’s disease pathology (*β* = −0.360, 95% CI: −0.598 to −0.121), Aβ load (*β* = −0.946, 95% CI: −1.712 to −0.179), and neurofibrillary tangles (*β* = −0.985, 95% CI: −1.831 to −0.140) (Table 3). There were no significant associations between PHD and the presence of chronic gross or microscopic infarcts, cerebral atherosclerosis, cerebral amyloid angiopathy, arteriolosclerosis, Lewy bodies, or hippocampal sclerosis.

**TABLE 3:** *β*‐Coefficients and odds ratios (ORs) with 95% confidence intervals (CIs) for the association between adherence to the Planetary Health Diet and brain pathologies: Results from linear and multivariable logistic regression models.

		Categorical (low as reference)	
Brain pathologies	Continuous	Moderate	High
Alzheimer's disease pathologies	*β* (95% CI)^a^	*β* (95% CI)^a^	*β* (95% CI)^a^
Global Alzheimer's disease pathology	−0.038 (−0.052, −0.025)	−0.317 (−0.537, −0.098)	−0.360 (−0.598, −0.121)
Amyloid beta load	−0.109 (−0.153, −0.065)	−0.612 (−1.621, −0.145)	−0.946 (−1.712, −0.179)
Neurofibrillary tangles	−0.113 (−0.162, −0.064)	−0.120 (−1.416, 0.192)	−0.985 (−1.831, −0.140)
Chronic infarcts	OR (95%CI)^a^	OR (95%CI)^a^	OR (95%CI)^a^
Gross infarcts	0.95 (0.83, 1.08)	1.06 (0.27, 4.21)	0.54 (0.13, 2.30)
Microscopic infarcts	0.99 (0.87, 1.14)	0.88 (0.22, 3.48)	1.03 (0.23, 4.53)
Vascular disease pathology	OR (95%CI)^a^	OR (95%CI)^a^	OR (95%CI)^a^
Cerebral atherosclerosis	0.99 (0.92, 1.09)	0.90 (0.28, 2.87)	0.85 (0.23, 3.09)
Cerebral amyloid angiopathy	0.98 (0.90, 1.07)	0.46 (0.13, 1.69)	0.57 (0.14, 2.33)
Arteriolosclerosis	0.98 (0.90, 1.06)	0.48 (0.15, 1.57)	0.72 (0.20, 2.58)
Lewy bodies	0.97 (0.88, 1.06)	0.87 (0.19, 4.04)	0.60 (0.13, 2.83)
Hippocampal sclerosis	0.95 (0.81, 1.11)	0.77 (0.60, 9.99)	1.02 (0.07, 13.95)

*Note*: Global Alzheimer's disease pathology, amyloid beta load, and neurofibrillary tangles were square root‐transformed.

Abbreviation: PHD, Planetary Health Diet.

^a^ Model adjusted for age at death, sex, education, *APOE ε*4, Mini‐Mental State Examination score, alcohol consumption, physical activity, body mass index, vascular disease burden (including claudication, stroke, heart conditions, and congestive heart failure), vascular risk factors (including hypertension, diabetes, and smoking history), multivitamin use, and total energy intake.

### 3.4 PHD and cognitive function proximate to death

Table 4 shows the association of the PHD with cognitive performance across several domains proximate to death before and after adjusting for global Alzheimer’s disease pathology. In Model A, which was adjusted for demographic, lifestyle, and health‐related factors, greater PHD adherence (as a continuous variable) was significantly associated with higher global cognitive scores (*β* = 0.011, 95% CI: 0.003–0.019) and better visuospatial ability (*β* = 0.015, 95% CI: 0.171–0.362). Participants with high compared to low PHD adherence showed significantly better performance in global cognition (*β* = 0.101, 95% CI: 0.013–0.188), episodic memory (*β* = 0.123, 95% CI: 0.008–0.239), and visuospatial ability (*β* = 0.116, 95% CI: 0.008–0.224).

**TABLE 4:** *β*‐Coefficients and 95% CIs for the association between adherence to the PHD and cognitive performance across multiple domains proximate to death before and after adjusting for global Alzheimer’s disease pathology: Results from linear regression models.

	Global cognition	Episodic memory	Semantic memory	Working memory	Visuospatial ability	Perceptual speed
PHD	*β* (95% CI)	*β* (95% CI)	*β* (95% CI)	*β* (95% CI)	*β* (95% CI)^a^	*β* (95% CI)^a^
Model A^a^						
Continuous	0.011 (0.003, 0.019)	0.010 (−0.002, 0.021)	0.015 (−0.003, 0.027)	0.007 (−0.004, 0.018)	0.015 (0.171, 0.362)	0.010 (−0.001, 0.022)
Categorical						
Low	Ref.	Ref.	Ref.	Ref.	Ref.	Ref.
Moderate	0.070 (−0.009, 0.150)	0.119 (−0.014, 0.225)	0.096 (−0.024 0.216)	0.006 (−0.111, 0.110)	0.049 (−0.049, 0.148)	0.045 (−0.070, 0.161)
High	0.101 (0.013, 0.188)	0.123 (0.008, 0.239)	0.121 (−0.010, 0.252)	0.054 (−0.068, 0.175)	0.116 (0.008, 0.224)	0.066 (−0.060, 0.193)
Model B^b^						
Continuous	0.015 (0.003, 0.027)	0.017 (0.001, 0.033)	0.014 (−0.003, 0.031)	0.011 (−0.005, 0.027)	0.017 (0.019, 0.032)	0.013 (−0.004, 0.030)
Categorical						
Low	Ref.	Ref.	Ref.	Ref.	Ref.	Ref.
Moderate	0.050 (−0.070, 0.171)	0.124 (−0.038, 0.285)	0.054 (−0.121, 0.229)	0.037 (−0.203, 0.128)	0.046 (−0.105, 0.198)	0.111 (−0.182, 0.164)
High	0.160 (0.030, 0.289)	0.208 (0.035, 0.382)	0.099 (−0.088, 0.285)	0.147 (−0.030, 0.325)	0.148 (0.015, 0.310)	0.122 (−0.064, 0.308)

^a^ Model A was adjusted for age, sex, education, *APOE ε*4, Mini‐Mental State Examination score, alcohol consumption, physical activity, body mass index, vascular disease burden (including claudication, stroke, heart conditions, and congestive heart failure), vascular risk factors (including hypertension, diabetes, and smoking), multivitamin use, and total energy intake.

^b^ Model B was additionally adjusted for global Alzheimer's disease pathology, which was square root‐transformed.

These associations remained robust after further adjustment for global Alzheimer’s disease pathology in Model B. Notably, participants with high PHD adherence continued to demonstrate significantly better performance in global cognition (*β* = 0.160, 95% CI: 0.030–0.289), episodic memory (*β* = 0.208, 95% CI: 0.035–0.382), and visuospatial ability (*β* = 0.148, 95% CI: 0.015–0.310). The associations for semantic memory, working memory, and perceptual speed were not statistically significant in either model.

### 3.5 Supplementary analysis

The results were not materially altered when we repeated the main analyses after: (1) using a competing risk model with death as the competing event (Table S8), (2) excluding participants who developed dementia within the first 3 years of follow‐up (Table S9), and (3) performing multiple imputations for missing values of covariates, including BMI, *APOE ε*4 status, and alcohol consumption (Table S10).

## 4 DISCUSSION

In this community‐based cohort study, high adherence to the EAT‐Lancet PHD was associated with lower risk and delayed onset of dementia, as well as lower levels of Alzheimer’s disease‐related neuropathological burden, including Aβ deposition and neurofibrillary tangle accumulation. PHD adherence was also associated with better cognitive performance, a finding partially explained by Alzheimer’s disease‐related neuropathology, indicating that additional biological mechanisms may contribute to the observed neuroprotective effects.

Although several studies have linked healthy dietary patterns, such as the Mediterranean, DASH, and MIND diets, to reduced dementia risk, evidence regarding the PHD remains scarce.^5^, ^6^ To date, only one study has examined the association between adherence to the PHD and incident dementia. A recent Swedish cohort study reported that higher adherence to the PHD was associated with a lower risk of AD, particularly among *APOE ε*4 noncarriers.^12^ Consistent with this, our findings showed that greater adherence to the PHD was associated with significantly lower risk and delayed onset of AD, reinforcing the potential cognitive benefits of this diet in a well‐characterized U.S. cohort with late‐life clinical follow‐up. When compared with other established dietary patterns, the PHD demonstrated comparable or even stronger protective associations with dementia risk, underscoring its potential as a sustainable dietary approach for cognitive health.

Our study takes a step beyond the current literature by examining the relationship between PHD and postmortem brain pathological data. We found that greater PHD adherence was associated with significantly lower global Alzheimer’s disease pathology, including Aβ load and neurofibrillary tangle burden. These results provide neurobiological plausibility for the observed dementia risk reduction and suggest that environmentally sustainable diets may also protect against the underlying neurodegenerative processes of Alzheimer’s disease. These results are in line with a previous investigation from MAP in which greater adherence to the MIND and Mediterranean diets was also associated with lower postmortem Alzheimer’s disease pathology, particularly reduced Aβ burden.^35^

Emerging studies have explored the relationship between PHD adherence and cognitive performance across diverse populations, but findings remain mixed.^16^, ^17^, ^18^ In the Gothenburg H70 Birth Cohort Study, PHD was not significantly associated with cognitive performance measures, including memory and executive function.^16^ In contrast, greater adherence to the PHD was linked to slower cognitive decline, particularly in executive function, among high‐income Brazilian adults in the ELSA‐Brasil study.^16^ Similarly, in a large Chinese cohort, associations were observed between higher PHD adherence and slower decline in both global cognition and memory‐specific domains.^18^ Domain‐specific associations were also reported in a Dutch study, with slower decline observed in executive functioning and attention among cognitively healthy older adults.^19^ Crucially, we extend previous work by integrating cognitive data with postmortem neuropathological data, revealing that the apparent protective effects of the PHD on cognition are partially explained by Alzheimer’s disease‐related pathology. This suggests that additional biological mechanisms, beyond Aβ and tau burden, may underlie the observed benefits.

The mechanisms behind the observed association between the PHD diet and cognitive health remain incompletely understood,^36^ and further studies are warranted to understand pathways beyond reduced AD pathology that may contribute. Diets rich in plant‐based foods and low in red meat and ultra‐processed items (such as the PHD) have been shown to reduce systemic inflammation, improve vascular function, and enhance insulin sensitivity, all of which are relevant to brain aging.^37^, ^38^, ^39^ Emerging evidence also points to the role of gut microbiota, which may influence neuroinflammation and amyloid processing via the gut–brain axis.^40^, ^41^, ^42^ Moreover, improved micronutrient intake and antioxidant status may protect against oxidative stress and neuronal damage.^43^, ^44^ Further experimental and longitudinal studies, including randomized trials and biomarker‐based research, are warranted to elucidate the biological pathways linking PHD adherence with neurodegeneration and cognitive aging.

Strengths of this study include the use of a well‐characterized community‐based cohort with a relatively large sample size, long follow‐up, and detailed clinical, cognitive, and postmortem brain pathological data. In addition, we used a validated FFQ to derive the EAT‐Lancet diet score, ensuring the reliability of the dietary data. Nonetheless, some limitations should be pointed out. First, the participants in MAP were volunteers, and were better educated and healthier than the general population,^45^ which may have contributed to an underestimation of the magnitude of the association between PHD and dementia risk. Moreover, data on brain pathology were captured only when the participants were deceased; thus, the causal inference between PHD and pathologies should be interpreted with caution. Another limitation is that the study sample comprised predominantly White volunteers who agreed to annual evaluations and postmortem organ donation, thus limiting the generalizability of findings to individuals of other races and ethnicities. Finally, although our analyses incorporated a variety of demographic, socioeconomic, and lifestyle‐related factors, we cannot rule out the possibility of residual confounding due to unmeasured factors.

In conclusion, our findings provide novel and compelling evidence that adherence to the PHD is associated with lower risk of dementia, attenuated global Alzheimer’s disease pathology (including Aβ and tau pathology), and preserved cognitive function across multiple domains near the end of life, even after adjusting for global Alzheimer’s disease pathology. These findings suggest that a sustainable dietary pattern may contribute to promoting healthy brain aging and provide a foundation for dietary strategies aimed at preserving cognitive resilience in older age.

## AUTHOR CONTRIBUTIONS

R.Y. and W.X. conceptualized and designed the study. R.Y. and J.W. conducted statistical analyses. R.Y. performed the literature search and drafted the manuscript. J.W., A.D., S.S., P.A., and D.A.B. interpreted the data and provided critical revisions to the manuscript. W.X. and D.A.B. supervised the study. All authors contributed substantially to the final version of the manuscript and approved it for publication.

## CONFLICT OF INTEREST STATEMENT

The authors report no disclosures relevant to the manuscript. Author disclosures are available in the supporting information.

## CONSENT STATEMENT

All participants provided written informed consent for these studies.

## Supporting information



Supporting Information

Supporting Information

## References

[1] Global status report on the public health response to dementia. https://www.who.int/publications/i/item/9789240033245 (accessed July 9, 2025)

[2] Livingston G , Huntley J , Liu KY , et al. Dementia prevention, intervention, and care: 2024 report of the Lancet standing Commission. Lancet 2024;404:572‐628. 39096926 10.1016/S0140-6736(24)01296-0

[3] Lulu W , Zili Z , Kobkullaya N , et al. Cognitively enhanced Tai Ji Quan: wisdom in a global promotion of traditional Chinese exercise. Acupunct Herb Med 2024;4(4):561‐562.

[4] Guasch‐Ferré M , Willett WC . The Mediterranean diet and health: a comprehensive overview. J Int Med 2021;290:549‐566. 10.1111/joim.13333 34423871

[5] Morris MC , Tangney CC , Wang Y , et al. MIND diet associated with reduced incidence of Alzheimer's disease. Alzheimers Dement 2015;11:1007‐1014. 25681666 10.1016/j.jalz.2014.11.009 PMC4532650

[6] Tangney CC , Li H , Wang Y , et al. Relation of DASH‐ and Mediterranean‐like dietary patterns to cognitive decline in older persons. Neurol 2014;83:1410‐1416. 10.1212/WNL.0000000000000884 PMC4206157 25230996

[7] Willett W , Rockström J , Loken B , et al. Food in the Anthropocene: the EAT–Lancet Commission on healthy diets from sustainable food systems. Lancet 2019;393:447‐492. 30660336 10.1016/S0140-6736(18)31788-4

[8] Knuppel A , Papier K , Key TJ , et al. EAT‐Lancet score and major health outcomes: the EPIC‐Oxford study. Lancet 2019;394:213‐214. 31235280 10.1016/S0140-6736(19)31236-X

[9] Sawicki CM , Ramesh G , Bui L , et al. Planetary Health Diet and cardiovascular disease: results from three large prospective cohort studies in the USA. Lancet Planetary Health 2024;8:e666‐74. 39243782 10.1016/S2542-5196(24)00170-0 PMC13739212

[10] Berthy F , Allès B , Fezeu LK , et al. Adherence to the EAT‐Lancet reference diet and risk of type 2 diabetes: results from the NutriNet‐Santé cohort study. Int J Epidemiol 2025;54:dyaf011. 40334153 10.1093/ije/dyaf011

[11] Dalile B , Kim C , Challinor A , et al. The EAT‐Lancet reference diet and cognitive function across the life course. Lancet Planetary Health 2022;6:e749‐59. 36087605 10.1016/S2542-5196(22)00123-1

[12] Samuelsson J , Glans I , Stubbendorff A , et al. Associations between the EAT‐Lancet Planetary Health Diet and incident dementia. J Prev Alzheimers Dis 2025;12:100166. 40222839 10.1016/j.tjpad.2025.100166 PMC12434276

[13] Morris MC , Tangney CC , Wang Y , et al. MIND diet slows cognitive decline with aging. Alzheimers Dement 2015;11:1015‐1022. 26086182 10.1016/j.jalz.2015.04.011 PMC4581900

[14] Jack CR , Bennett DA , Blennow K , et al. NIA‐AA Research Framework: toward a biological definition of Alzheimer's disease. Alzheimers Dement 2018;14:535‐562. 29653606 10.1016/j.jalz.2018.02.018 PMC5958625

[15] Sperling RA , Aisen PS , Beckett LA , et al. Toward defining the preclinical stages of Alzheimer's disease: recommendations from the National Institute on Aging‐Alzheimer's Association workgroups on diagnostic guidelines for Alzheimer's disease. Alzheimers Dement 2011;7:280‐292. 21514248 10.1016/j.jalz.2011.03.003 PMC3220946

[16] Gomes Gonçalves N , Cacau LT , Ferreira NV , et al. Adherence to the Planetary Health Diet and cognitive decline: findings from the ELSA‐Brasil study. Nat Aging 2024;4:1465‐1476. 38942982 10.1038/s43587-024-00666-4

[17] Samuelsson J , Stubbendorff A , Marseglia A , et al. A comparative study of the EAT‐Lancet diet and the Mediterranean diet in relation to neuroimaging biomarkers and cognitive performance. Alzheimers Dement 2025;21:e70191. 40302043 10.1002/alz.70191 PMC12040733

[18] Tang L , Yu X , Qiu C , et al. Adherence to the Planetary Health Diet is associated with slower cognitive decline: a prospective cohort analysis of Chinese older adults. Int J Behav Nutr Phys Act 2025;22:56. 40380205 10.1186/s12966-025-01759-y PMC12082918

[19] van Soest APM , van de Rest O , Witkamp RF , et al. The association between adherence to the EAT‐Lancet diet and cognitive ageing. Age Ageing 2024;53:ii39‐46. 38745489 10.1093/ageing/afae032 PMC11094393

[20] Bennett DA , Schneider JA , Buchman AS , et al. Overview and findings from the Rush Memory and Aging Project. Curr Alzheimer Res 2012;9:646‐663. 22471867 10.2174/156720512801322663 PMC3439198

[21] Morris MC , Tangney CC , Bienias JL , et al. Validity and reproducibility of a food frequency questionnaire by cognition in an older biracial sample. Am J Epidemiol 2003;158:1213‐1217. 14652307 10.1093/aje/kwg290

[22] Agarwal P , Agrawal S , Wagner M , et al. MIND diet and hippocampal sclerosis among community‐based older adults. JAMA Netw Open 2025;8:e2526089. 40773193 10.1001/jamanetworkopen.2025.26089 PMC12332637

[23] Stubbendorff A , Sonestedt E , Ramne S , Drake I , Hallström E , Ericson U . Development of an EAT‐Lancet index and its relation to mortality in a Swedish population. Am J Clin Nutr 2022;115:705‐716. 34791011 10.1093/ajcn/nqab369 PMC8895215

[24] Barnes LL , Dhana K , Liu X , et al. Trial of the MIND diet for prevention of cognitive decline in older persons. N Engl J Med 2023;389:602‐611. 37466280 10.1056/NEJMoa2302368 PMC10513737

[25] Wilson RS , Boyle PA , Yu L , et al. Temporal course and pathologic basis of unawareness of memory loss in dementia. Neurol 2015;85:984‐991. 10.1212/WNL.0000000000001935 PMC4567465 26311746

[26] Bennett DA , Schneider JA , Aggarwal NT , et al. Decision rules guiding the clinical diagnosis of Alzheimer's disease in two community‐based cohort studies compared to standard practice in a clinic‐based cohort study. Neuroepidemiol 2006;27:169‐176. 10.1159/000096129 17035694

[27] Dhana K , Agarwal P , James BD , et al. Healthy lifestyle and cognition in older adults with common neuropathologies of dementia. JAMA Neurol 2024;81:233‐239. 38315471 10.1001/jamaneurol.2023.5491 PMC10845037

[28] Song R , Xu H , Dintica CS , et al. Associations between cardiovascular risk, structural brain changes, and cognitive decline. J Am Coll Cardiol 2020;75:2525‐2534. 32439001 10.1016/j.jacc.2020.03.053 PMC10061875

[29] Wilson RS , Boyle PA , Yu L , et al. Life‐span cognitive activity, neuropathologic burden, and cognitive aging. Neurol 2013;81:314‐321. 10.1212/WNL.0b013e31829c5e8a PMC3772831 23825173

[30] Bennett DA , Schneider JA , Arvanitakis Z , et al. Neuropathology of older persons without cognitive impairment from two community‐based studies. Neurol 2006;66:1837‐1844. 10.1212/01.wnl.0000219668.47116.e6 16801647

[31] Boyle PA , Yu L , Leurgans SE , et al. Attributable risk of Alzheimer's dementia attributed to age‐related neuropathologies. Ann Neurol 2019;85:114‐124. 30421454 10.1002/ana.25380 PMC10128614

[32] Bennett DA , Schneider JA , Wilson RS , et al. Neurofibrillary tangles mediate the association of amyloid load with clinical Alzheimer disease and level of cognitive function. Arch Neurol 2004;61:378‐384. 15023815 10.1001/archneur.61.3.378

[33] Arvanitakis Z , Leurgans SE , Barnes LL , et al. Microinfarct pathology, dementia, and cognitive systems. Stroke 2011;42:722‐727. 21212395 10.1161/STROKEAHA.110.595082 PMC3042494

[34] Wang J , Dove A , Song R , et al. Poor pulmonary function is associated with mild cognitive impairment, its progression to dementia, and brain pathologies: a community‐based cohort study. Alzheimers Dement 2022;18:2551‐2559. 35184372 10.1002/alz.12625 PMC10078691

[35] Agarwal P , Leurgans SE , Agrawal S , et al. Association of Mediterranean‐DASH Intervention for Neurodegenerative Delay and Mediterranean Diets with Alzheimer Disease Pathology. Neurol 2023;100:e2259‐e2268. doi:10.1212/WNL.0000000000207176 10.1212/WNL.0000000000207176 PMC10259273 36889921

[36] Townsend RF , Logan D , O'Neill RF , et al. Whole dietary patterns, cognitive decline and cognitive disorders: a systematic review of prospective and intervention studies. Nutrients 2023;15:333. 36678204 10.3390/nu15020333 PMC9865080

[37] Satija A , Bhupathiraju SN , Rimm EB , et al. Plant‐based dietary patterns and incidence of type 2 diabetes in US men and women: results from three prospective cohort studies. PLOS Med 2016;13:e1002039. 27299701 10.1371/journal.pmed.1002039 PMC4907448

[38] Satija A , Bhupathiraju SN , Spiegelman D , et al. Healthful and unhealthful plant‐based diets and the risk of coronary heart disease in U.S. adults. J Am Coll Cardiol 2017;70:411‐422. 28728684 10.1016/j.jacc.2017.05.047 PMC5555375

[39] Del Bo’ C , Chehade L , Tucci M , et al. Impact of substituting meats with plant‐based analogues on health‐related markers: a systematic review of human intervention studies. Nutrients 2024;16:2498. 39125378 10.3390/nu16152498 PMC11314210

[40] Li B , He Y , Ma J , et al. Mild cognitive impairment has similar alterations as Alzheimer's disease in gut microbiota. Alzheimers Dement 2019;15:1357‐1366. 31434623 10.1016/j.jalz.2019.07.002

[41] Schaible P , Henschel J , Erny D . How the gut microbiota impacts neurodegenerative diseases by modulating CNS immune cells. J Neuroinflammation 2025;22:60. 40033338 10.1186/s12974-025-03371-0 PMC11877772

[42] Cryan JF , O'Riordan KJ , Cowan CSM , et al. The microbiota‐gut‐brain axis. Physiol Rev 2019;99:1877‐2013. 31460832 10.1152/physrev.00018.2018

[43] Joseph JA , Shukitt‐Hale B , Casadesus G . Reversing the deleterious effects of aging on neuronal communication and behavior: beneficial properties of fruit polyphenolic compounds2, 3. Am J Clin Nutr 2005;81:313S‐316S. 15640496 10.1093/ajcn/81.1.313S

[44] Vauzour D , Camprubi‐Robles M , Miquel‐Kergoat S , et al. Nutrition for the ageing brain: towards evidence for an optimal diet. Ageing Res Rev 2017;35:222‐240. 27713095 10.1016/j.arr.2016.09.010

[45] Wang S , Wang J , Dove A , et al. association of impaired kidney function with dementia and brain pathologies: a community‐based cohort study. Alzheimers Dement 2023;19:2765‐2773. 36571791 10.1002/alz.12910 PMC12139445

