# Supplementary material for: Association of the Planetary Health Diet with dementia risk and brain pathology
Source: Alzheimers Dement. 2026 Apr 1;22(4):e71327. doi: 10.1002/alz.71327 (PMC13045258; doi:10.1002/alz.71327)
Supplement: Supplementary file 1 — Supporting Information [file ALZ-22-e71327-s001.docx]

**Supplemental Materials**

**Supplementary Method 1** Assessment of cognitive function

**Supplementary Method 2** Assessment of postmortem brain pathology

**Supplementary Method 3** Assessment of other covariates

**Supplementary table 1** Description of the food components included in the EAT-Lancet diet score

**Supplementary table 2** Criteria for the EAT-Lancet diet score

**Supplementary table 3.** Differences in median time to dementia and Alzheimer’s dementia onset across levels of adherence to the Planetary Health Diet, estimated using Laplace regression models.

**Supplementary table 4.** Association between adherence to the Planetary Health Diet and dementia and Alzheimer’s dementia by age.

**Supplementary table 5.** Association between adherence to the Planetary Health Diet and dementia and Alzheimer’s dementia by sex.

**Supplementary table 6.** Association between adherence to the Planetary Health Diet and dementia and Alzheimer’s dementia by APOE ε4 status.

**Supplementary table 7.** Hazard ratios (HRs) and 95% confidence intervals (CIs) for the associations of adherence to the MIND, Mediterranean, and Dietary Approaches to Stop Hypertension (DASH) dietary patterns with risk of all-cause dementia: results from Cox regression models.

**Supplementary table 8.** Association between adherence to the Planetary Health Diet and dementia and Alzheimer’s dementia using competing risk models.

**Supplementary table 9.** Association between adherence to the Planetary Health Diet and risk of dementia and Alzheimer’s dementia after excluding participants who developed dementia within the first three years of follow-up.

**Supplementary table 10.** Association between adherence to the Planetary Health Diet and dementia and Alzheimer’s dementia with multiple imputations for missing data.

**Supplementary Figure 1.** Flow chart for the study population.

**References**

**Supplementary Method 1**

**Assessment of Cognitive Function**

Cognitive function was assessed using a battery of 19 standardized tests covering five domains.^1^ Episodic memory was evaluated with seven cognitive tests: word list memory, word list recall, word list recognition, and immediate and delayed recall from the East Boston Story and Story A from Logical Memory. Semantic memory was assessed using three cognitive tests: Boston Naming, category fluency, and the National Adult Reading Test. Working memory was evaluated using three cognitive tests: Digits Forward, Digits Backward, and Digit Ordering. Visuospatial ability was tested using Judgement of Line Orientation and the Standard Progressive Matrices. Perceptual speed was assessed using four cognitive tests: the oral version of the Symbol Digit Modalities Test, Number Comparison, and two indices from a modified Stroop Neuropsychological Screening Test.

**Supplementary Method 2**

**Assessment of postmortem Brain Pathology**

**Cerebral atherosclerosis**

Atherosclerosis was evaluated by visual inspection of the vertebral, basilar, posterior cerebral, middle cerebral, and anterior cerebral arteries and their proximal branches and was rated using a semiquantitative scale with 7 levels from no atherosclerosis to severe involvement of most vessels or more than 75% occlusion of one or more vessels.^2^

**Arteriolosclerosis**

Arteriolosclerosis refers to the histological changes in the small vessels of the brain that are responsible for narrowing the vascular lumen. Vessels of the anterior basal ganglia were evaluated with a semiquantitative scaling system from 0 (none) to 7 (occluded).^3^

**Macroscopic cerebral infarcts (macroinfarcts)**

Cerebral infarcts visible to the naked eye on fixed slabs were examined and documented the age, volume (in cubic millimeters), side, and location (side and region) of infarcts visible to the naked eye. Chronic macroinfarcts were coded as present or absent and included in the analyses.^4^

**Microscopic cerebral infarcts (microinfarcts)**

Microinfarcts were defined as any infarct seen only by microscopic examination on a minimum of nine regions in one hemisphere on 6μm paraffin-embedded sections stained with hematoxylin/eosin. The following regions were examined: six cortical regions (midfrontal, middle temporal, entorhinal, hippocampal, inferior parietal, and anterior cingulate cortices), two subcortical regions (anterior basal ganglia, thalamus), and midbrain.^5^

**Cerebral amyloid angiopathy**

Cerebral amyloid angiopathy pathology is assessed in 4 neocortical regions: midfrontal, midtemporal, parietal, and calcarine cortices. Meningeal and parenchymal vessels were assessed for amyloid deposition for each region and scored from 0 to 4. Cerebral amyloid angiopathy score for each region was the maximum of the meningeal and parenchymal. Scores were averaged across regions and summarized as a continuous measure of cerebral amyloid angiopathy pathology.^6^

**Lewy body disease pathology**

Lewy bodies were assessed in 6 regions, including substantia nigra, anterior cingulate cortex, entorhinal cortex, midfrontal cortex, superior or middle temporal cortex, inferior parietal cortex using a monoclonal phosphorylated antibody to α-synuclein (1:20,000; Wako Chemical, Richmond, VA) with alkaline phosphatase as the chromogen. Details for the diagnosis of Lewy body we have previously described.^7^

**Hippocampal sclerosis**

Hippocampal sclerosis was evaluated unilaterally in a coronal section of the midhippocampus at the level of the lateral geniculate body, and graded as absent or present based on severe neuronal loss and gliosis in CA1 and/or subiculum as described previously.^8^

**Supplementary Method 3**

**Assessment of** **other covariates**

Age at death was computed from dates of birth and death. Sex was self-reported using U.S. census questions. Education was self-reported as the number of years of formal schooling completed. *Apolipoprotein E* (APOE) genotype was determined by the Broad Institute for Population Genetics using the Polymorphic DNA Technologies using the Polymorphic DNA Technologies.^9^ Participants were classified as APOE e4 carriers if they had 1 or both (i.e., 2) e4 alleles or noncarriers if they had none.^10^ Body mass index was calculated as weight (kg) divided by the square of height (m^2^). Alcohol consumption was obtained from the FFQ and expressed as the average amount of alcohol (grams per day) consumed over the previous year. Physical activity was operationalized as participants’ total number of hours of physical activity per week based on the National Health Interview Survey.^11^ Vascular disease burden was calculated based on self-reported history of four major conditions: intermittent claudication, stroke, coronary heart disease, and congestive heart failure. Vascular risk factors were defined as the presence of hypertension, diabetes mellitus, and current or past history of smoking.^11^ The Mini–Mental State Examination (MMSE) is a widely used 30-item standardized tool for assessing global cognitive function and dementia severity. It has been incorporated into numerous epidemiologic studies and is a core component of the CERAD (Consortium to Establish a Registry for Alzheimer’s Disease) neuropsychological battery. The MMSE has high short-term temporal stability and shows strong correlations with other dementia severity scales. The test evaluates multiple cognitive domains, including orientation to time and place, immediate and delayed recall, attention and calculation (e.g., serial sevens or spelling "WORLD" backwards), language abilities, and vasoconstriction.

**Supplementary table 1. Description of the food components included in the EAT-Lancet diet score.**

| **Food components** | **Food items** |
| --- | --- |
| **Emphasized food** | |
| Vegetables | All vegetables such as broccoli, spinach, green/red peppers, Lettuce/tossed salad and except. |
| Fruits | All fruits such as raisins, grapes, bananas, cantaloupe melons apples, pears, oranges, grapefruit, strawberries, and peaches. |
| Legumes | Dry beans, lentils, peas, soy. Targets and index refer to raw weight. |
| Whole grains | Cold breakfast cereal, whole wheat bread / brown bread, and other whole grains like kasha and  quinoa. Targets and index refer to raw weight. |
| Nuts | All nuts and seeds including peanuts and nut mixes. |
| Fish | Fish, fish products, Shrimp, lobster, and scallops. |
| Unsaturated oils | Mono-and polyunsaturated fatty acid content in foods |
| **Limited food** | |
| Beef and lamb | Beef, lamb, minced meat with pork and lamb, processed meat with beef and lamb. |
| Pork | Pork, minced meat of pork, processed meat with pork. |
| Poultry | Chicken, turkey, and other poultry. |
| Eggs | Eggs. |
| Dairy | Whole milk, low-fat milk, skim milk, yoghurt and other fermented milk products, cheese, and cream. |
| Potatoes | Mashed potatoes, potato salad, yams, and potatoes included in dishes. |
| Added sugar | Sucrose and monosaccharides except sugars in fruits and vegetables. |

**Supplementary table 2. Criteria for the EAT-Lancet score.**

| **Food components** | |  | **Target intake (reference interval)**  **Mean gram intake/day** | **3 points**  **Mean gram intake/day** | **2 points**  **Mean gram intake/day** | **1 point**  **Mean gram intake/day** | **0 points**  **Mean gram intake/day** |
| --- | --- | --- | --- | --- | --- | --- | --- |
| **Emphasized intake** | Vegetables | | 300 (200–600) | >300 | 200–300 | 100–200 | <100 |
|  | Fruits | | 200 (100–300) | >200 | 100–200 | 50–100 | <50 |
|  | Unsaturated oils | | 40 (20–80) | >40 | 20–40 | 10–20 | <10 |
|  | Legumes | | 75 (0–150) | >75 | 37.5–75 | 18.75–37.5 | <18.75 |
|  | Nuts | | 50 (0–100) | >50 | 25–50 | 12.5–25 | <12.5 |
|  | Whole grains | | 232 (total grains 0-60% of energy) | >232 | 116–232 | 58–116 | <58 |
|  | Fish | | 28 (0–100) | >28 | 14–28 | 7–14 | <7 |
| **Limited intake** | Beef and lamb | | 7 (0–14) | <7 | 7–14 | 14–28 | >28 |
|  | Pork | | 7 (0–14) | <7 | 7–14 | 14–28 | >28 |
|  | Poultry | | 29 (0–58) | <29 | 29–58 | 58–116 | >116 |
|  | Eggs | | 13 (0–25) | <13 | 13–25 | 25–50 | >50 |
|  | Dairy | | 250 (0–500) | <250 | 250–500 | 500–1000 | >1000 |
|  | Potatoes | | 50 (0–100) | <50 | 50–100 | 100–200 | >200 |
|  | Added sugar | | 31 (0–31) | <31 | 31–62 | 62–124 | >124 |

**Supplementary table 3.** Differences in median time to dementia and Alzheimer’s dementia onset across levels of adherence to the Planetary Health Diet, estimated using Laplace regression models.

| **Planetary Health Diet** | **Dementia** |  | **Alzheimer’s dementia** | |
| --- | --- | --- | --- | --- |
|  | **Difference in**  **median age ^a^** | **Difference in**  **median age ^b^** | **Difference in**  **median age ^a^** | **Difference in**  **median age ^b^** |
| Continuous | 0.12 (0.01, 0.24) | 0.20 (0.04, 0.35) | 0.18 (0.04, 0.33) | 0.22 (0.08, 0.35) |
| Categorical |  |  |  |  |
| Low | Ref. | Ref. | Ref. | Ref. |
| Moderate | 1.10 (-0.47, 2.66) | 1.56 (0.31, 2.81) | 1.25 (-0.24, 2.74) | 1.89 (0.76, 3.02) |
| High | 2.01 (0.18, 3.84) | 2.17 (0.54, 3.79) | 1.98 (0.26, 3.69) | 2.51 (1.09, 3.93) |

*APOE*, *apolipoprotein E*; MMSE, Mini Mental State Examination; BMI, body mass index.

The number of subjects with missing values was 17 (1.84%) for BMI, 5 (0.54%) for *APOE* ε4, and 7 (0.76%) for alcohol consumption.

^a^ Adjusted for age, sex, education, and total energy intake.

^b^ Model adjusted for age, sex, education, *APOE* ε4, MMSE score, alcohol consumption, physical activity, BMI, vascular disease burden (including claudication, stroke, heart conditions and congestive heart failure), vascular risk factors including (hypertension, diabetes and smoking), multivitamin use, and total energy intake.

**Supplementary table 4.** Association between adherence to the Planetary Health Diet and dementia and Alzheimer’s dementia by age.

| **Planetary Health Diet** | **Dementia** | | |  | **Alzheimer’s dementia** | | |
| --- | --- | --- | --- | --- | --- | --- | --- |
|  | **No. of cases** | **HR (95% CI) ^a^** | **HR (95% CI) ^b^** |  | **No. of cases** | **HR (95% CI) ^a^** | **HR (95% CI) ^b^** |
| **Age < 80** |  |  |  |  |  |  |  |
| Continuous | 121 | 0.97 (0.93-1.01) | 0.98 (0.94-1.02) |  | 117 | 0.97 (0.93-1.00) | 0.98 (0.94-1.00) |
| Categorical (tertiles) | |  |  |  |  |  |  |
| Low | 43 | Ref. | Ref. |  | 42 | Ref. | Ref. |
| Moderate | 41 | 0.73 (0.47-1.13) | 0.78 (0.50-1.11) |  | 40 | 0.73 (0.47-1.14) | 0.78 (0.50–1.21) |
| High | 37 | 0.67 (0.43-1.07) | 0.74 (0.47-0.99) |  | 35 | 0.66 (0.41-1.07) | 0.69 (0.45-0.98) |
| **Age ≥ 80** |  |  |  |  |  |  |  |
| Continuous | 190 | 0.96 (0.93-0.99) | 0.96 (0.93-0.99) |  | 184 | 0.96 (0.92-0.99) | 0.95 (0.91-0.99) |
| Categorical (tertiles) | |  |  |  |  |  |  |
| Low | 82 | Ref. | Ref. |  | 81 | Ref. | Ref. |
| Moderate | 64 | 0.79 (0.57-1.11) | 0.77 (0.55-1.10) |  | 60 | 0.75 (0.53-1.06) | 0.73 (0.51-1.03) |
| High | 44 | 0.65 (0.44-0.95) | 0.65 (0.43-0.96) |  | 43 | 0.65 (0.44-0.95) | 0.64 (0.43-0.96) |

*APOE*, *apolipoprotein E*; MMSE, Mini Mental State Examination; BMI, body mass index.

The number of subjects with missing values was 17 (1.84%) for BMI, 5 (0.54%) for *APOE* ε4, and 7 (0.76%) for alcohol consumption.

^a^ Adjusted for sex, education, and total energy intake.

^b^ Model adjusted for sex, education, *APOE* ε4, MMSE score, alcohol consumption, physical activity, BMI, vascular disease burden (including claudication, stroke, heart conditions and congestive heart failure), vascular risk factors including (hypertension, diabetes and smoking), multivitamin use, and total energy intake.

**Supplementary table 5.** Association between adherence to the Planetary Health Diet and dementia and Alzheimer’s dementia by sex.

| **Planetary Health Diet** | **Dementia** | | |  | **Alzheimer’s dementia** | | |
| --- | --- | --- | --- | --- | --- | --- | --- |
|  | **No. of cases** | **HR (95% CI) ^a^** | **HR (95% CI) ^b^** |  | **No. of cases** | **HR (95% CI) ^a^** | **HR (95% CI) ^b^** |
| **Male** |  |  |  |  |  |  |  |
| Continuous | 70 | 0.96 (0.90-1.01) | 0.98 (0.92-1.04) |  | 67 | 0.98 (0.92-1.03) | 0.98 (0.93-1.04) |
| Categorical (tertiles) | |  |  |  |  |  |  |
| Low | 20 | Ref. | Ref. |  | 18 | Ref. | Ref. |
| Moderate | 32 | 0.89 (0.51-1.55) | 0.95 (0.74-2.38) |  | 32 | 0.98 (0.55-1.74) | 0.97 (0.82–2.31) |
| High | 18 | 0.71 (0.38-1.35) | 0.89 (0.47-0.99) |  | 17 | 0.75 (0.39-1.45) | 0.91 (0.50-0.99) |
| **Female** |  |  |  |  |  |  |  |
| Continuous | 241 | 0.97 (0.95-1.00) | 0.97 (0.94-0.99) |  | 234 | 0.97 (0.95-1.00) | 0.96 (0.93-0.99) |
| Categorical (tertiles) | |  |  |  |  |  |  |
| Low | 105 | Ref. | Ref. |  | 105 | Ref. | Ref. |
| Moderate | 73 | 0.78 (0.57-1.06) | 0.75 (0.55-1.03) |  | 68 | 0.73 (0.52-1.00) | 0.70 (0.51-0.96) |
| High | 63 | 0.73 (0.53-1.02) | 0.71 (0.51-0.99) |  | 61 | 0.71 (0.51-0.99) | 0.69 (0.49-0.97) |

*APOE*, *apolipoprotein E*; MMSE, Mini Mental State Examination; BMI, body mass index.

The number of subjects with missing values was 17 (1.84%) for BMI, 5 (0.54%) for *APOE* ε4, and 7 (0.76%) for alcohol consumption.

^a^ Adjusted for age, education and total energy intake.

^b^ Model adjusted for age, education, *APOE* ε4, MMSE score, alcohol consumption, physical activity, BMI, vascular disease burden (including claudication, stroke, heart conditions and congestive heart failure), vascular risk factors including (hypertension, diabetes and smoking), multivitamin use, and total energy intake.

**Supplementary table 6.** Association between adherence to the Planetary Health Diet and dementia and Alzheimer’s dementia by *APOE* ε4 status.

| **Planetary Health Diet** | **Dementia** | | |  | **Alzheimer’s dementia** | | |
| --- | --- | --- | --- | --- | --- | --- | --- |
|  | **No. of cases** | **HR (95% CI) ^a^** | **HR (95% CI) ^b^** |  | **No. of cases** | **HR (95% CI) ^a^** | **HR (95% CI) ^b^** |
| **APOE ε4 carriers** | |  |  |  |  |  |  |
| Continuous | 85 | 0.99 (0.95-1.04) | 0.96 (0.95-1.03) |  | 85 | 0.99 (0.96-1.05) | 0.98 (0.95-1.00) |
| Categorical (tertiles) | |  |  |  |  |  |  |
| Low | 31 | Ref. | Ref. |  | 29 | Ref. | Ref. |
| Moderate | 29 | 0.82 (0.46-1.27) | 0.85 (0.45-1.32) |  | 29 | 0.86 (0.48-1.37) | 0.90 (0.47-1.41) |
| High | 28 | 0.76 (0.50-1.09) | 0.77 (0.49-1.00) |  | 27 | 0.81 (0.50-1.50) | 0.82 (0.51-1.00) |
| **APOE ε4 non-carriers** | |  |  |  |  |  |  |
| Continuous | 223 | 0.96 (0.93-0.99) | 0.96 (0.93-0.99) |  | 216 | 0.95 (0.92-0.98) | 0.96 (0.92-0.99) |
| Categorical (tertiles) | |  |  |  |  |  |  |
| Low | 95 | Ref. | Ref. |  | 95 | Ref. | Ref. |
| Moderate | 74 | 0.79 (0.57-1.08) | 0.79 (0.58-1.08) |  | 69 | 0.73 (0.53-1.00) | 0.73 (0.53-1.01) |
| High | 54 | 0.66 (0.46-0.94) | 0.70 (0.49-0.99) |  | 52 | 0.63 (0.44-0.90) | 0.67 (0.47-0.96) |

*APOE*, *apolipoprotein E*; MMSE, Mini Mental State Examination; BMI, body mass index.

The number of subjects with missing values was 17 (1.84%) for BMI, 5 (0.54%) for *APOE* ε4, and 7 (0.76%) for alcohol consumption.

^a^ Adjusted for age, sex, education, and total energy intake.

^b^ Model adjusted for age, sex, education, MMSE score, alcohol consumption, physical activity, BMI, vascular disease burden (including claudication, stroke, heart conditions and congestive heart failure), vascular risk factors including (hypertension, diabetes and smoking), multivitamin use, and total energy intake.

**Supplementary table 7.** Hazard ratios (HRs) and 95% confidence intervals (CIs) for the associations of adherence to the MIND, Mediterranean, and Dietary Approaches to Stop Hypertension (DASH) dietary patterns with risk of all-cause dementia: results from Cox regression models.

| **Diet** | **All-cause dementia** | | |
| --- | --- | --- | --- |
|  | **No. of cases** | **HR (95% CI) ^a^** | **HR (95% CI) ^b^** |
| **MIND diet** |  |  |  |
| Continuous | 311 | 0.90 (0.84-0.97) | 0.92 (0.85-1.00) |
| Categorical (tertiles) |  |  |  |
| Low | 123 | Ref. | Ref. |
| Moderate | 105 | 0.73 (0.56-0.95) | 0.70 (0.53-0.91) |
| High | 83 | 0.69 (0.52-0.91) | 0.73 (0.54-0.99) |
| **Mediterranean diet** |  |  |  |
| Continuous | 311 | 0.96 (0.94-0.99) | 0.97 (0.94-0.99) |
| Categorical (tertiles) |  |  |  |
| Low | 118 | Ref. | Ref. |
| Moderate | 111 | 0.69 (0.53-0.90) | 0.66 (0.50-0.86) |
| High | 82 | 0.66 (0.49-0.89) | 0.67 (0.49-0.92) |
| **DASH diet** |  |  |  |
| Continuous | 311 | 0.95 (0.87-1.03) | 0.95 (0.87-1.04) |
| Categorical (tertiles) |  |  |  |
| Low | 143 | Ref. | Ref. |
| Moderate | 84 | 0.88 (0.67-1.15) | 0.88 (0.67-1.16) |
| High | 84 | 0.79 (0.60-1.03) | 0.81 (0.61-1.07) |

^a^ Adjusted for age, sex, education, and total energy intake.

^b^ Model adjusted for age, sex, education, *apolipoprotein E* ε4, Mini Mental State Examination score, alcohol consumption, physical activity, body mass index, vascular disease burden (including claudication, stroke, heart conditions and congestive heart failure), vascular risk factors including (hypertension, diabetes and smoking), multivitamin use, and total energy intake.

**Supplementary table 8.** Association between adherence to the Planetary Health Diet and dementia and Alzheimer’s dementia using competing risk models.

| **Planetary Health Diet** | **Dementia** |  |  | **Alzheimer’s dementia** | |
| --- | --- | --- | --- | --- | --- |
|  | **HR (95% CI) ^a^** | **HR (95% CI) ^b^** |  | **HR (95% CI) ^a^** | **HR (95% CI) ^b^** |
| Continuous | 0.97 (0.95-0.99) | 0.97 (0.95-1.00) |  | 0.97 (0.94-0.99) | 0.97 (0.94-0.99) |
| Categorical |  |  |  |  |  |
| Low | Ref. | Ref. |  | Ref. | Ref. |
| Moderate | 0.86 (0.66-1.12) | 0.84 (0.64-1.10) |  | 0.82 (0.63-1.08) | 0.81 (0.61-1.06) |
| High | 0.76 (0.57-1.01) | 0.76 (0.57-0.99) |  | 0.76 (0.57-1.01) | 0.75 (0.56-0.99) |

*APOE*, *apolipoprotein E*; MMSE, Mini Mental State Examination; BMI, body mass index.

The number of subjects with missing values was 17 (1.84%) for BMI, 5 (0.54%) for *APOE* ε4, and 7 (0.76%) for alcohol consumption.

^a^ Adjusted for age, sex, education, and total energy intake.

^b^ Model adjusted for age, sex, education, *APOE* ε4, MMSE score, alcohol consumption, physical activity, BMI, vascular disease burden (including claudication, stroke, heart conditions and congestive heart failure), vascular risk factors including (hypertension, diabetes and smoking), multivitamin use, and total energy intake.

**Supplementary table 9.** Association between adherence to the Planetary Health Diet and risk of dementia and Alzheimer’s dementia after excluding participants who developed dementia within the first three years of follow-up.

| **Planetary Health Diet** | **Dementia** | | |  | **Alzheimer’s dementia** | | |
| --- | --- | --- | --- | --- | --- | --- | --- |
|  | **No. of cases** | **HR (95% CI) ^a^** | **HR (95% CI) ^b^** |  | **No. of cases** | **HR (95% CI) ^a^** | **HR (95% CI) ^b^** |
| Continuous | 223 | 0.99 (0.96-1.02) | 0.98 (0.95-0.99) |  | 218 | 0.98 (0.96-1.00) | 0.96 (0.93-0.99) |
| Categorical | (tertiles) |  |  |  |  |  |  |
| Low | 72 | Ref. | Ref. |  | 72 | Ref. | Ref. |
| Moderate | 81 | 0.94 (0.70-1.28) | 0.92 (0.69-1.17) |  | 78 | 0.91 (0.67-1.18) | 0.85 (0.53-0.99) |
| High | 70 | 0.83 (0.73-1.04) | 0.81 (0.63-0.98) |  | 68 | 0.98 (0.71-0.99) | 0.95 (0.68-0.97) |

*APOE*, *apolipoprotein E*; MMSE, Mini Mental State Examination; BMI, body mass index.

^a^ Adjusted for age, sex, education, and total energy intake.

^b^ Model adjusted for age, sex, education, *APOE* ε4, MMSE score, alcohol consumption, physical activity, BMI, vascular disease burden (including claudication, stroke, heart conditions and congestive heart failure), vascular risk factors including (hypertension, diabetes and smoking), multivitamin use, and total energy intake.

**Supplementary table 10.** Association between adherence to the Planetary Health Diet and dementia and Alzheimer’s dementia with multiple imputations for missing data.

| **Planetary Health Diet** | **Dementia** |  | **Alzheimer’s dementia** |
| --- | --- | --- | --- |
|  | **HR (95% CI) ^a^** |  | **HR (95% CI) ^a^** |
| Continuous | 0.97 (0.94-0.99) |  | 0.97 (0.94-0.99) |
| Categorical (tertiles) |  |  |  |
| Low | Ref. |  | Ref. |
| Moderate | 0.79 (0.60-1.02) |  | 0.74 (0.59-0.99) |
| High | 0.72 (0.54-0.96) |  | 0.69 (0.51-0.93) |

*APOE*, *apolipoprotein E*; MMSE, Mini Mental State Examination; BMI, body mass index.

Imputed variables included BMI (17, 1.84%), APOE ε4 status (5, 0.54%), and alcohol consumption (7, 0.76%).

^a^ Model adjusted for age, sex, education, *APOE* ε4, MMSE score, alcohol consumption, physical activity, BMI, vascular disease burden (including claudication, stroke, heart conditions and congestive heart failure), vascular risk factors including (hypertension, diabetes and smoking), multivitamin use, and total energy intake.

**
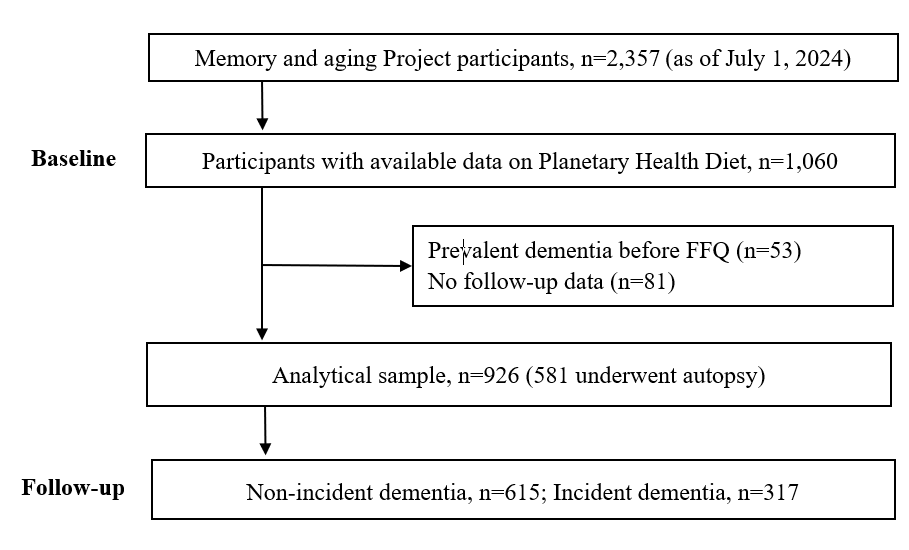
**

**Supplementary Figure 1.** Flow chart for the study population.

FFQ, food frequency questionnaire.

**Reference**

1 Wang J, Song R, Dove A, *et al.* Pulmonary Function is Associated with Cognitive Decline and Structural Brain Differences. *Alzheimers Dement* 2022; **18**: 1335–44.

2 Arvanitakis Z, Capuano AW, Leurgans SE, Buchman AS, Bennett DA, Schneider JA. The Relationship of Cerebral Vessel Pathology to Brain Microinfarcts. *Brain Pathology* 2017; **27**: 77–85.

3 Cerebrovascular Disease Pathology and Parkinsonian Signs in Old Age | Stroke. https://www-ahajournals-org.proxy.kib.ki.se/doi/10.1161/STROKEAHA.111.623462?url_ver=Z39.88-2003&rfr_id=ori:rid:crossref.org&rfr_dat=cr_pub%20%200pubmed (accessed July 12, 2025).

4 The Apolipoprotein E ε4 Allele Increases the Odds of Chronic Cerebral Infarction Detected at Autopsy in Older Persons | Stroke. https://www-ahajournals-org.proxy.kib.ki.se/doi/10.1161/01.STR.0000160747.27470.2a?url_ver=Z39.88-2003&rfr_id=ori:rid:crossref.org&rfr_dat=cr_pub%20%200pubmed (accessed July 12, 2025).

5 Arvanitakis Z, Leurgans SE, Barnes LL, Bennett DA, Schneider JA. Microinfarct pathology, dementia, and cognitive systems. *Stroke* 2011; **42**: 722–7.

6 Cerebral amyloid angiopathy and cognitive outcomes in community-based older persons | Neurology. https://www-neurology-org.proxy.kib.ki.se/doi/10.1212/WNL.0000000000002175?url_ver=Z39.88-2003&rfr_id=ori:rid:crossref.org&rfr_dat=cr_pub%20%200pubmed (accessed July 12, 2025).

7 Schneider JA, Arvanitakis Z, Yu L, Boyle PA, Leurgans SE, Bennett DA. Cognitive impairment, decline and fluctuations in older community-dwelling subjects with Lewy bodies. *Brain* 2012; **135**: 3005–14.

8 Nag S, Yu L, Capuano AW, *et al.* Hippocampal sclerosis and TDP-43 pathology in aging and Alzheimer disease. *Annals of Neurology* 2015; **77**: 942–52.

9 Bennett DA, Wilson RS, Schneider JA, *et al.* Apolipoprotein E ε4 allele, AD pathology, and the clinical expression of Alzheimer’s disease. *Neurology* 2003; **60**: 246–52.

10 Bennett DA, Schneider JA, Buchman AS, Mendes de Leon C, Bienias JL, Wilson RS. The Rush Memory and Aging Project: Study Design and Baseline Characteristics of the Study Cohort. *Neuroepidemiology* 2005; **25**: 163–75.

11 Song R, Xu H, Dintica CS, *et al.* Associations Between Cardiovascular Risk, Structural Brain Changes, and Cognitive Decline. *Journal of the American College of Cardiology* 2020; **75**: 2525–34.
